# Interkingdom signaling elicited by bacterial extracellular vesicles in human cystic fibrosis airway epithelium and neutrophils

**DOI:** 10.3389/fcimb.2026.1695102

**Published:** 2026-03-02

**Authors:** Navya Lakkappa, Deepali Luthra, Prashant Kumar, Eloy Almenar Perez, Jennifer Hynes, Paul McNally, Fiona Ringholz, Basil El Nazir, Georgia Bateman, Aoife M. Rodgers, Kelsey Brew, Shuichi Takayama, Clifford C. Taggart, Rabindra Tirouvanziam, Judith A. Coppinger

**Affiliations:** 1 School of Pharmacy and Biomolecular Sciences, Royal College of Surgeons in Ireland (RCSI) University of Medicine and Health Sciences, Dublin, Ireland; 2 Cystic Fibrosis Centre and Translational Research Centre, Children’s Health Ireland (CHI), Dublin, Ireland; 3 Department of Pediatrics, Emory University, and Center of CF & Airways Disease Research, Children’s Healthcare of Atlanta, Atlanta, GA, United States; 4 Department of Paediatrics, Royal College of Surgeons in Ireland (RCSI) University of Medicine and Health Sciences, Dublin, Ireland; 5 Department of Paediatrics, Trinity College Dublin, Dublin, Ireland; 6 Airway Innate Immunity Research (AiiR) Group, Wellcome-Wolfson Institute for Experimental Medicine and Wellcome-Wolfson Institute for Experimental Medicine, School of Medicine, Dentistry and Biomedical Sciences, Queen’s University Belfast, Belfast, United Kingdom; 7 Wallace H. Coulter Department of Biomedical Engineering, The Georgia Institute of Technology and Emory University, Atlanta, GA, United States

**Keywords:** bacteria, cystic fibrosis, epithelium, extracellular vesicles, neutrophils

## Abstract

**Rationale:**

Defects in epithelial host defense and dysfunctional neutrophilic inflammation in the presence of sustained bacterial infection underpin chronic lung disease in cystic fibrosis (CF). Deciphering mechanisms on how bacteria interact with the host to establish chronic infection is critical for disease control in people with CF (pwCF).

**Objective:**

We sought to investigate the co-existence of microbial and host extracellular vesicles (EVs) in CF airway fluids and evaluate if bacterial EVs from *Pseudomonas* (*P.*) *aeruginosa* impact EV release by host cells (epithelia and neutrophils) from pwCF.

**Methods:**

EVs from sputum and bronchoalveolar lavage fluid from pwCF (*n* = 26 samples total) were characterized by nanoparticle tracking analysis (NTA) and mass spectrometry (MS)-based proteomics. The impact of EVs from *P. aeruginosa* (wild-type and select mutant strains) on functional responses of human epithelial cultures derived from pwCF and control subjects (*n* = 8) and human neutrophils transmigrated into CF and control lung conditions (*n* = 3) was examined using NTA and MS, and flow cytometry, respectively.

**Results:**

We observed the co-existence of bacterial and host EVs in airway fluid from pwCF. Functionally, exposure to EVs from wild-type *P. aeruginosa* impacted the number and content of host EVs released from epithelia of pwCF and the surface phenotype of lung-conditioned neutrophils. Furthermore, mutations modifying the content of *P. aeruginosa* EVs impacted host epithelial EV release and neutrophil phenotype.

**Conclusion:**

This proof-of-concept study suggests host–bacterial interplay via EVs in CF airways.

## Introduction

Concomitant neutrophil-driven inflammation and bacterial infection define lung disease in human cystic fibrosis (CF). Gaining novel insights into how bacteria interact with the host to establish chronic infection under the sustained presence of neutrophils is critical, as persistent areas of inflammation and infection eventually lead to loss of lung function and respiratory failure in people with CF (pwCF) (Gangell et al., 2011; Tang et al., 2014). Prior studies from our group suggest that, from the host perspective, neutrophils, macrophages, and epithelia in CF airways undergo profound functional reprogramming, leading to an active host tolerance state toward bacteria (Tirouvanziam et al., 2008; Laval et al., 2013; Margaroli et al., 2021). From the microbial perspective, bacteria acquired from the environment and otherwise known as pathogens, including *Staphylococcus* (*S.*) *aureus* and *Pseudomonas* (*P.*) *aeruginosa*, are selected and maintained in a quasi-commensal relationship with the host, losing virulence factors in the process (Gabrielaite et al., 2020). Re-energizing bacterial clearance in CF airways without making the host more prone to lung damage by host proteases and oxidases, or reactivating virulence pathways in bacteria, is a therapeutic challenge (Giacalone et al., 2020). Greater insights into how bacteria communicate with host cells are needed. For example, commensals may modulate host immunity by suppressing pro-clearance responses elicited upon infection (Stanton, 2021).

Emerging research in chronic lung diseases has demonstrated a role for both host and microbial extracellular vesicles (EVs) in modulating host immune response to microbial challenges. EVs are present in all human biofluids such as plasma, urine, saliva, and airway fluid and include exosomes, microvesicles, ectosomes, and apoptotic vesicles, ranging from 50 to 1,000 nm (Théry et al., 2018). Human biofluids may also contain microbial EVs, which have evolved along host EVs to carry out functions ranging from induction of cell migration into mucosal tissues to the intercellular transfer of proteins, RNAs, and metabolites, which can induce cell activation or inhibition (Trappe et al., 2021; Wahlund et al., 2017; Mathieu et al., 2019). Prior studies by our group and others have demonstrated that host EVs derived from epithelial cells and neutrophils can promote pro-inflammatory signaling and contribute to antibacterial responses in CF (Genschmer et al., 2019; Useckaite et al., 2020; Forrest et al., 2022; Trappe et al., 2023). For example, the secretion of EVs from human bronchial cells was shown to play an important role in modulating the immune response to *P. aeruginosa* lung infection (Sarkar et al., 2024).

Several groups have also shown the ability of microbial EVs to elicit host immune responses through binding of pathogen-associated molecular patterns (PAMPs) to pattern recognition receptors like Toll-like receptors (TLRs) on epithelia (Mancini et al., 2020; Bomberger et al., 2009) and macrophages (Cecil et al., 2017). This can result in the secretion of pro-inflammatory cytokines (notably IL-8), which in turn can drive the continuous recruitment of neutrophils to the CF airway lumen. Additionally, bacterial EVs have shown promise in the regulation of immune response and pathogenicity. For example, targeting a regulatory RNA in EVs was shown to reduce the epithelial innate immune response (Koeppen et al., 2016). Bacterial EVs have also been shown to enhance biofilm formation (Yonezawa et al., 2009), and this may in turn influence EV signaling, as EVs isolated from *P. aeruginosa* PA14 biofilms induced lower bactericidal response compared to EVs from planktonic PA14 cultures (Cooke et al., 2019).

Here, we demonstrate the co-existence of microbial EVs and host EVs in CF airway fluid and investigate interkingdom crosstalk elicited by the former. Specifically, we show that EVs secreted by *P. aeruginosa* can impact the number and content of EVs released from CF airway cultures upon co-incubation. Furthermore, we show that selectively modifying the content of *P. aeruginosa* EVs impacts EV release by human airway epithelial cells and the activation profile of lung-recruited neutrophils.

## Methods

We provide below essential methodological information related to experiments conducted in the study. Detailed methods related to immunoblotting, flow cytometry, and electron microscopy, which were performed as previously published (Shields et al., 2018; Useckaite et al., 2020; Forrest et al., 2022), are available in online **Supplementary Figures**.

### Human samples

Bronchoalveolar lavage fluid (BALF) and brushings were obtained through the SHIELD-CF (Study of Host Immunity and Early Lung Disease in Cystic Fibrosis) and RECOVER (Real World Clinical Outcomes with Novel Modulator Therapy Combinations in People with CF) studies among children with CF at the Children’s Health Ireland (CHI) and adults with CF attending St. Vincent’s University Hospital (SVUH), respectively (McNally et al., 2023). Donors for sputum and BALF were heterozygous or homozygous for Phe508del. All study participants were recruited as approved by the Ethics Medical Research Committee at CHI or SVUH. Bronchial brushings were obtained via consent from persons with CF (with different CFTR mutations) and control donors (non-CF persons undergoing respiratory tests) via bronchoscopy with CHI ethics approval (GEN/807/20; GEN/228/11). CF blood for neutrophil isolation was obtained under an approved IRB protocol at Emory University (IRB0042577).

### Epithelial cell culture

Cells from bronchial brushings were obtained as previously reported (Ringholz et al., 2017), with the following adaptations. In brief, cells were pelleted at 500*g* and resuspended in PneumaCult™-Ex plus medium (STEMCELL Technologies, Vancouver, Canada) supplemented with 50 µg/mL of penicillin–streptomycin, 50 µg/mL of gentamicin, and 1.25 µg/mL of amphotericin B. Resuspended cells were seeded into a human collagen-coated T25 flask and incubated at 37 °C in 5% CO_2_ in PneumaCult™-Ex plus medium, until reaching 70% confluency. Then, cells were trypsinized, counted, and seeded at a density of 50 × 10^4^ cells/mL onto the apical compartment of 6.5 mm CELLTREAT^®^ 0.4 μm polyethylene membrane inserts (STEMCELL Technologies, Vancouver, Canada) containing PneumaCult™-Ex plus medium in both apical and basal compartments. The cells were assessed every 2 days for confluence by measuring transepithelial electrical resistance (TEER) until it reached 800–1,000 Ω, which occurred at approximately 13–14 days. Subsequently, cultures were switched to air–liquid interface (ALI) by exposing the apical surface to air, and basal media was switched to PneumaCult™-ALI basal medium (STEMCELL Technologies, Vancouver, Canada), supplemented with 50 µg/mL of penicillin–streptomycin, 50 µg/mL of gentamicin, and 1.25 µg/mL of amphotericin B.

### EV isolation

EVs were isolated from different types of clinical samples, including BALF and sputum, as well as primary cultures from patient bronchial brushings. For BALF and sputum, this involved centrifuging samples at 500*g* for 10 min to remove cells and large debris, followed by ultracentrifuging twice at 120,000*g* for 120 min (XL-70 ultracentrifuge, Beckman Coulter, Brea, CA, United States). For cultures, 200 µL of media was added to the apical chamber to collect EVs. Supernatants from both apical and basal chambers were collected, centrifuged at 500*g* for 10 min to remove cell debris, and ultracentrifuged twice at 120,000*g* for 120 min at 4°C to pellet EVs.

### EV collection from *P. aeruginosa* and *S. aureus* cultures

*P. aeruginosa* (PA01) and *S. aureus* (USA300 MRSA) strains were obtained and streaked on Luria–Bertani (LB) agar plates. Mutated strains of *P. aeruginosa* were obtained from the sequence-verified two-allele transposon mutant library from the Salipante group, as previously described (Held et al., 2012). Specifically, along with wild-type (WT) PA01, mutant strains with mutations in the outer membrane porin F (OprF−), arginine deaminase (ADI−), and arginine decarboxylase (ADC−) genes were used. Bacteria were cultured to exponential phases and EVs isolated from culture media as previously reported (Zare Banadkoki et al., 2022) and outlined in the **Supplementary Methods**.

### Epithelial cell exposure to bacterial EVs

A safe dose of EVs for treatment of ALI cultures was determined by the MTT assay. Nanoparticle tracking analysis (NTA) was also used to analyze the number of EVs in each dilution. Based on viability data, ALI cultures were treated apically with 0.676 × 10^8^ EVs/mL and incubated for 24 h, after which their basal and apical supernatants were collected and frozen until further analysis.

### IL-8 quantification in epithelial cultures

Thawed basal and apical supernatants of epithelial cultures were centrifuged at 500*g* for 10 min to remove cell debris. Clear supernatants were diluted 1:10, and IL-8 was quantified using the AuthentiKine™ human IL-8 ELISA kit (Proteintech, Chicago, IL, United States) following the manufacturer’s protocol.

### Lung conditioning and bacterial EV exposure of human neutrophils

*In vitro* lung conditioning of neutrophils was conducted by transmigrating primary blood neutrophils as described before (Margaroli et al., 2021), using the modified 96-well endothelial/epithelial co-culture model (Viola et al., 2025). Briefly, to ensure the integrity of the endothelial (HUVEC) and epithelial (H441) cell barriers, TEER values were recorded across wells. Neutrophils were isolated from the blood of CF donors, as described previously (Forrest et al., 2022), and placed on the endothelium for transendothelial and transepithelial migration toward either of three conditions: CF airway supernatant (CFASN, cell-free sputum product, a pathological fluid containing chemoattractants and both host and bacterial EVs), leukotriene 4 (LTB4, 100 nM, used as a chemoattractant control), or media (negative control) placed on the apical surface of the epithelium. Isolated blood neutrophils were used as pre-transmigration controls. As experimental conditions for transmigration, EVs isolated from *P. aeruginosa* PA01 WT and mutants (OprF−, ADI−, ADC−) were placed on the apical surface of the epithelium (with 100 nM of LTB4 to promote neutrophil transmigration). Neutrophils were collected 14 h post-transmigration and stained for flow cytometry with antibodies against CD11b, CD16, CD62L, CD63, CD66b, and the Live/Dead zombie NIR dye (all from BioLegend, San Diego, CA, United States). Flow cytometry data were acquired using an Aurora spectral flow cytometer (Cytek Biosciences, Fremont, CA, United States), and results were analyzed using FlowJo v.9.9.5 (BD Biosciences, Franklin Lakes, NJ, United States).

### EV mass spectrometry-based proteomics

**Figure 1:**
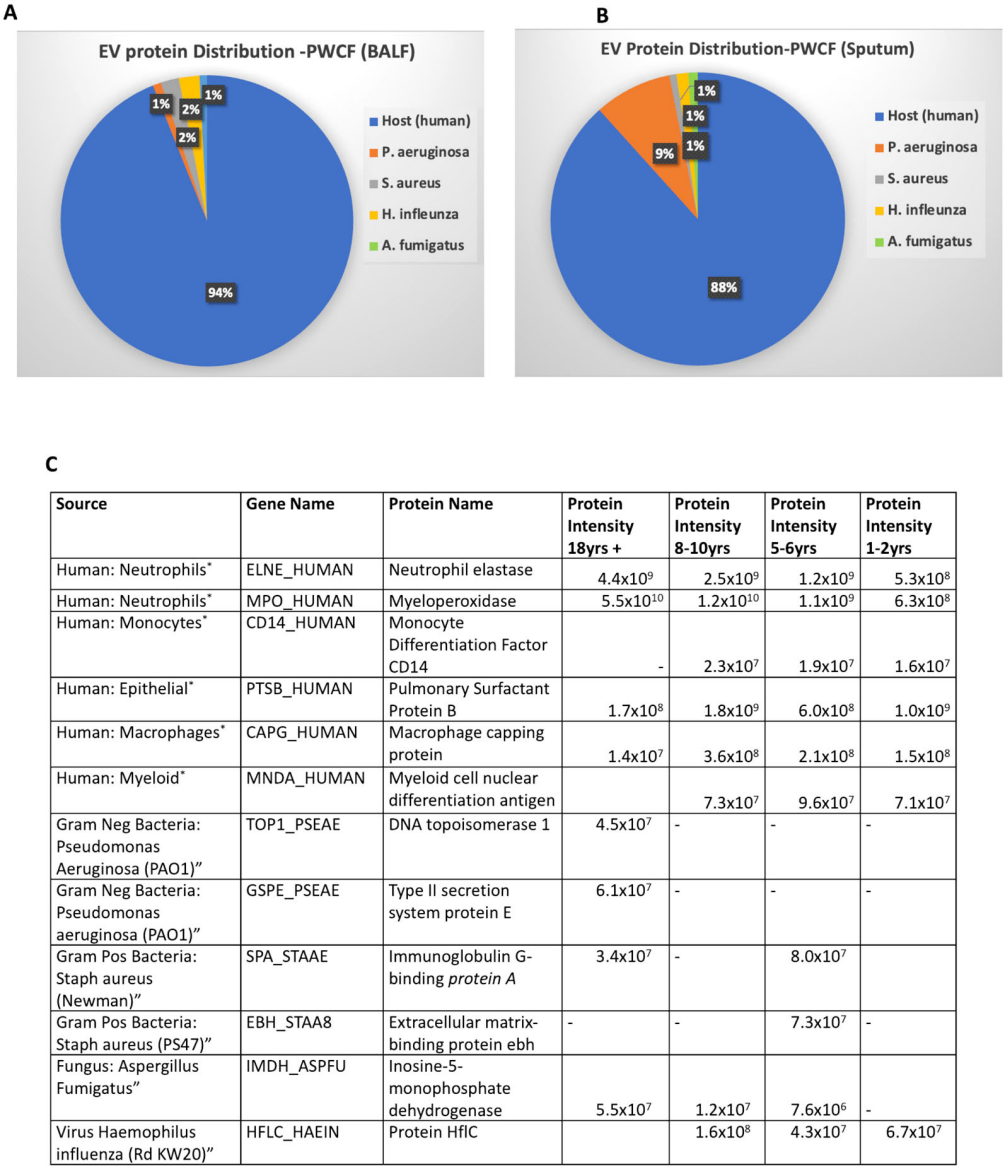
Host and microbial EV proteins co-exist in CF airways. **(A)** MS-based spectra generated from BALF EVs from pwCF (*n* = 16) were searched using human and microbial protein databases. The percentage of proteins identified by MS in the BALF EVs is displayed using Venny software per species (relative to total proteins identified). **(B)** Sputum EVs obtained from pwCF (*n* = 10) and subjected to MS analysis were searched against human and microbial protein databases as above. The percentage of proteins identified by MS in the sputum EVs is displayed per species using Venny software. **(C)** The table highlights 12 of the EV proteins identified in BALF (**Supplementary Table 2**) from five species. Normalized label-free quantification (LFQ) intensities (protein intensities) generated by MaxQuant for 12 EV proteins in BALF and sputum are displayed along with the gene and protein names. *Most reported source but proteins may have more than one cell source.

For proteomic analysis, EVs isolated from samples were resuspended in 8 M of urea, reduced, alkylated, and subjected to tryptic digestion. Samples were run on a Bruker TimsTOF Pro mass spectrometer connected to an Evosep One chromatography system, as previously described (Trappe et al., 2023). Raw data were searched against the *Homo sapiens* subset of the UniProt/Swiss-Prot database using MaxQuant (https://www.maxquant.org/, release 2.6.3.0) with specific parameters for trapped ion mobility spectra data-dependent acquisition (TIMS DDA). Additionally, spectra were searched using the MaxQuant algorithm against reviewed protein sequenced databases from UniProtKB for four common CF infectious agents, namely, *P. aeruginosa*, *S. aureus*, *Haemophilus* (*H.*) *influenzae*, and *Aspergillus* (*A.*) *fumigatus*, for both BALF and sputum EVs (**Figure 1**). EVs derived from patient-derived cultures were searched against the *H. sapiens* database. Label-free quantification (LFQ) intensities were generated by MaxQuant for EV proteins. LFQ intensity values are a measure of the relative protein abundance across different samples that have been searched together and normalized. Identified proteins were analyzed using Gene Ontology (GO) enrichment analysis from the Gene Ontology website, which connects to the Panther Classification system, and via ShinyGO, a graphical gene enrichment tool that links gene sets to functional pathways and cellular processes (Ge et al., 2020). LFQ was used to compare the relative abundance of given proteins across samples (Cox et al., 2014).

### Statistical analysis

Analyses were conducted using Prism 10 (GraphPad Software, San Diego, CA, United States). Paired comparisons of pre- vs. post-bacterial EV exposure conditions used a non-parametric Wilcoxon rank test. A non-parametric Kruskal–Wallis test was used for analyses featuring three or more groups.

## Results

### EVs from host cells and microbes co-exist in CF airway fluid

To probe the co-existence of EVs from host and microbial origin in the airway fluid of pwCF, EVs were isolated from CF airway fluids, BALF, and sputum. EV size was confirmed by NTA (**Supplementary Figure 1**). Mass spectrometry (MS) based proteomic and bioinformatic analysis was then performed on digested sputum samples (*n* = 10) and a BALF (*n* = 16) dataset (Useckaite et al., 2020) to detect the presence of microbial proteins. All donors were heterozygous or homozygous. The Phe508del and subject characteristics including microbial data are detailed in **Supplementary Table 1**. MS spectra were searched using the MaxQuant algorithm against reviewed protein sequenced databases from UniProt including *H. sapiens*, *P. aeruginosa*, *S. aureu*s*, A. fumigatus*, and *H. influenzae*. These four organisms commonly infect airways in CF (Renwick et al, 2014) and were identified by clinical records in pwCF (**Supplementary Table 1**).

The number of proteins identified by MS in the EVs from BALF (**Figure 1A**) and sputum (**Figure 1B**) is displayed per species using Venny software. In BALF, 94% of total proteins identified in EVs originated from the human host, 2% from *H. influenzae*, 2% from *S. aureus*, 1% from *A. fumigatus*, and 1% from *P. aeruginosa* (**Figure 1A**). In sputum, 88% of total proteins identified in EVs originated from the human host, 1% from *H. influenzae*, 1% from *S. aureus*, 1% from *A. fumigatus*, and 9% from *P. aeruginosa* (**Figure 1B**). The higher prevalence of *P. aeruginosa* proteins among EVs from sputum is likely attributable to the fact that sputum samples were sourced from teenagers and adults, whereas 12 out of 16 BALF samples were from children under 11 (**Supplementary Table 1**). The full list of proteins identified in BALF and sputum samples is included in **Supplementary Table 2**. Twelve representative proteins and their corresponding LFQ intensities from airway fluids of pwCF are displayed in **Figure 1C**. This includes six proteins of non-host origin, e.g., type 3 secretion protein originating from *P. aeruginosa*. The six host proteins among the 12 listed include neutrophil elastase and myeloperoxidase (predominantly made by neutrophils) and monocyte differentiating factor (restricted to monocytes).

### Exposure to *P. aeruginosa* EVs alters apical EV concentration in pwCF

**Figure 2:**
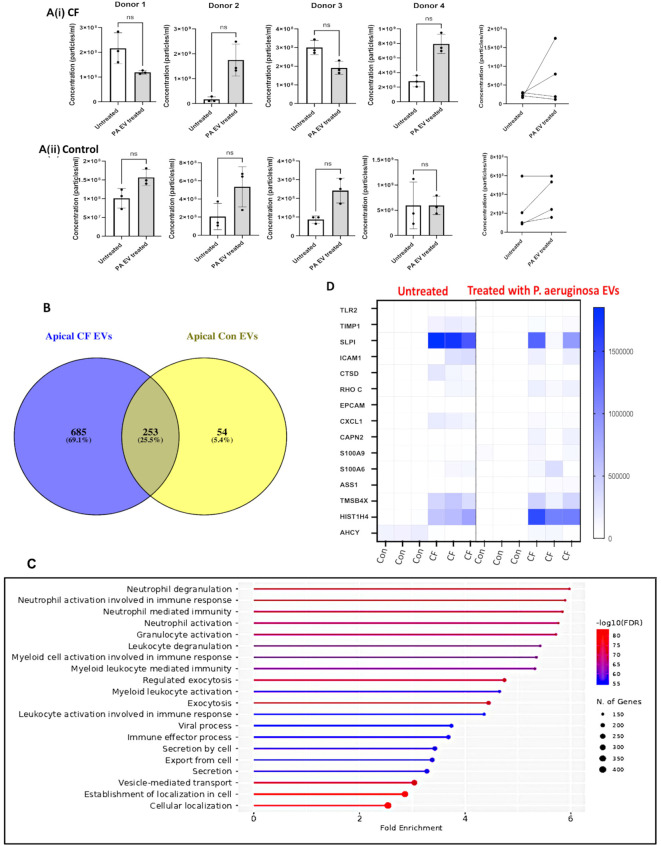
Airway cultures stimulated with *P. aeruginosa* EVs demonstrated changes in EV concentration and unique protein fingerprints compared to controls. The concentration of apical EV particles/mL pre-/post-*P. aeruginosa* EV treatment is displayed in **(A)** (i) for four separate pwCF and (ii) controls. Statistical tests used were paired non-parametric *t*-tests (Wilcoxon rank), and error bars denote means ± SD. **(B)** EVs were subjected to MS analysis **as before**. The number of EV proteins identified for CF vs. control is displayed using the Venn diagram software (Venny 2.10). **(C)** Significantly enriched GO biological processes (ShinyGO) for apical EV proteins from pwCF (treated with *P. aeruginosa* EVs) are displayed along with corresponding gene number and FDR (inverse log) range. **(D)** A heatmap illustrating LFQ intensities for 15 apical EV proteins (see **Supplementary Table 3**) significantly upregulated in pwCF pre- and post*-**P. aeruginosa*** EV treatment is displayed.

To probe interkingdom signaling via EVs, we exposed primary human airway epithelia to EVs from *P. aeruginosa* and assessed their own production of EVs in response. To do so, we first isolated EVs from *P. aeruginosa* cultures using ultracentrifugation as previously performed (Bomberger et al., 2009; Held et al., 2012). We confirmed the size of the *P. aeruginosa* EVs as being below 200 nm by NTA and confirmed the presence of OprF, a critical protein in EV biogenesis, by immunoblotting (**Supplementary Figure 2**). Next, airway epithelial cultures derived from bronchial brushings from pwCF and control donors were established. Tight junctions were fully formed at TEER of ~800–1,000 Ω, and further differentiation into a mucociliary layer was confirmed by staining for MUC5AC (**Supplementary Figure 3**). To stimulate epithelial airway cultures from control subjects and pwCF with *P. aeruginosa* EVs, we first conducted a dose–response experiment and ensured optimal epithelial cell viability with the MTT assay (**Supplementary Figure 4**), leading us to select a dose of 0.676 × 10^8^ *P. aeruginosa* EVs/mL. After exposure, epithelial EVs were isolated from both apical and basal compartments and validated by TEM and immunoblotting (**Supplementary Figure 5**). We observed patient-dependent changes in the levels of apical EV produced with an increase observed in half of the pwCF donors. Individual patient bar charts pre- vs. post-*P. aeruginosa* EV exposure are shown for pwCF [**Figure 2A**(i)] and control donors [**Figure 2A**(ii)]. Basal EV levels post-*P. aeruginosa* EV stimulation are shown in **Supplementary Figure 6**. The main observations we observed were as follows: 1) The average number of apical EVs was higher from four pwCF (7.1 × 10^8^ particles/mL) compared to control donors (3.8 × 10^8^) post-PA EV exposure; 2) there was a patient-dependent response in pwCF to *P. aeruginosa* EVs in particular for apical EVs [**Figures 2A**(i); and **3**] there were no significant changes in apical or basal EV size distribution profiles following *P. aeruginosa* EV exposure (**Supplementary Figure 7**).

Next, apical EVs of airway cultures from pwCF and control donors were isolated and subjected to MS-based proteomics. A higher number of proteins were identified in the former than the latter (*N* = 938 vs. *N* = 307 proteins, **Figure 2B**). Full annotation of these proteins is provided in **Supplementary Table 3**. Significantly enriched GO biological processes identified with Shingo for the apical EV proteins from cultures of pwCF donors upon exposure to *P. aeruginosa* EVs are listed in **Figure 2C**. An enrichment in neutrophil-mediated processes and vesicle transport was observed in both GO and REACTOME analyses (**Supplementary Table 3**, tabs 2 and 3). A heatmap illustrating LFQ intensities for 15 apical EV proteins upregulated in cultures of pwCF compared to control donors (pre- vs. post*-P. aeruginosa* EV exposure) includes proteins involved in CF and leukocyte recruitment (i.e., SLPI, ICAM-1; **Figure 2D**). We noted a significant increase in expression of several proteins involved in leukocyte responses in EVs from cultures of pwCF donors, regardless of exposure. We also observed differences in apical EVs comparing pre- vs. post-*P. aeruginosa* EV exposure in cultures of pwCF donors, with candidate proteins including SS1, arginosuccinate synthase, and histone H4.

### MS-based proteomics of bacterial EVs reveals modifiable signaling pathways

**Figure 3:**
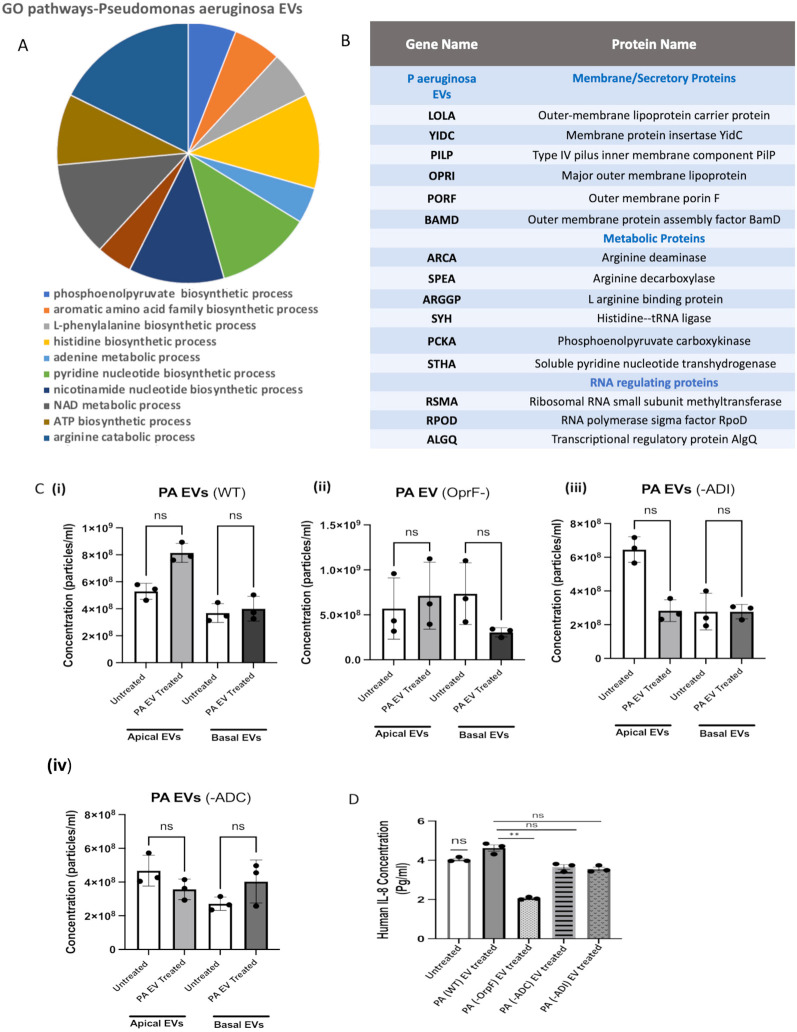
*P. aeruginosa* EVs are enriched in metabolic pathways and secretory, which can be modified to alter epithelial airway secretion in CF. **(A)** EVs from bacterial clinical strains were isolated and subjected to MS-based proteomics. The spectra were searched using the MaxQuant algorithm against a reviewed “*P. aeruginosa*” protein sequence database (UniProt). **(A)** The identified protein was analyzed using GO enrichment analysis performed on the GO home page (https://geneontology.org/docs/go-enrichment-analysis) connected to the Panther classification system. The top 10 enriched biological processes from *P. aeruginosa* are listed in a pie chart. **(B)** A table of 15 *P. aeruginosa* EV proteins. **(C)** EVs were isolated from WT (PAO1) and mutated strains (−OprF, −ADC, −ADI) of *P. aeruginosa*. The resulting secretion from apical EV and basal cultures was measured using NTA. The number of particles per mL of EVs in airway cultures incubated with WT *P. aeruginosa* EVs is displayed in [**(C)**(i)]. The number of particles per mL of EVs in airway cultures incubated with *P. aeruginosa* EVs derived from mutated strains with deletions in the following genes is displayed by bar chart (*n* = 3) with OprF displayed in [**(C)**(ii)], arginine deaminase (ADI−) in [**(C)**(iii)], and arginine decarboxylase (ADC−) in [**(C)**(iv)]. **(D)** The levels of IL-8 secretion under the above conditions were analyzed by ELISA, displayed as bar charts. Non-parametric Kruskal–Wallis statistical tests were used for all the above analyses featuring three or more groups.

EVs isolated from *P. aeruginosa* and *S. aureus* strains were subjected to MS analysis. For *P. aeruginosa* EVs, 809 proteins were identified (listed in **Supplementary Table 4**) and analyzed for significantly enriched GO biological processes. Metabolic processes were found enriched in the *P. aeruginosa* EV proteome (**Supplementary Table 4**, tab 2), with arginine and histidine metabolic pathways strongly represented (**Figure 3A**). Fifteen proteins identified in *P. aeruginosa* EVs are listed in **Figure 3B**. Comparatively, a smaller number of proteins (95) were identified in *S. aureus* EVs, with enriched biological processes listed in **Supplementary Figure 8**. Given the prevalence of *P. aeruginosa* in the CF sputum samples and the larger number of protein targets identified by MS in *P. aeruginosa* EVs, we applied a deeper focus to *P. aeruginosa* in further functional analyses.

Next, we set out to test the effects of EVs from strains of *P. aeruginosa* mutated in pathways associated with EV and arginine metabolism on epithelial cells. We selected mutant strains with a deletion in OprF and with mutations in two arginine metabolic enzymes, arginine deaminase (ADI) and arginine decarboxylase (ADC), from a two-allele transposon mutant *P. aeruginosa* library (University of Washington) and isolated their respective EVs. While the OprF− mutant is compromised in an EV adhesion protein, ADI− and ADC− strains display distinct changes in arginine metabolic enzymes in *P. aeruginosa* EVs (**Figure 3A**) and are also interesting considering the significance of arginine metabolism in CF airways (Grasemann et al., 2006). In addition to the original validation of sequence mutations by Illumina sequencing provided by the library, we confirmed the absence of OprF in EVs from the OprF− strain by Western blot (**Supplementary Figure 9**). Unfortunately, however, no antibodies were available for ADI and ADC proteins to conduct similar validation.

Epithelial EV secretion in apical and basal aspects of primary CF epithelial cultures (created from brushing from children with CF) pre- vs. post-*P. aeruginosa* EV exposure was assessed by NTA for WT [**Figure 3C**(i)], OprF− [**Figure 3C**(ii)], ADI− [**Figure 3C**(iii)], and ADC− [**Figure 3C**(iv)]. A decrease in epithelial EV secretion was observed upon exposure to EVs from the ADI− strain. Finally, IL-8 secretion by CF epithelial cultures under the above conditions was also analyzed by ELISA, and a significant decrease was observed post-exposure to EVs from *P. aeruginosa* mutant strains (OprF–) compared to wild-type (**Figure 3E**). We repeated these experiments using an adult CF cell model with similar trends observed, particularly with the levels of IL-8 post-*P. aeruginosa* EV exposure (**Supplementary Figure 10a–e**).

### Exposure to *P. aeruginosa* EVs impacts lung neutrophil activation

**Figure 4:**
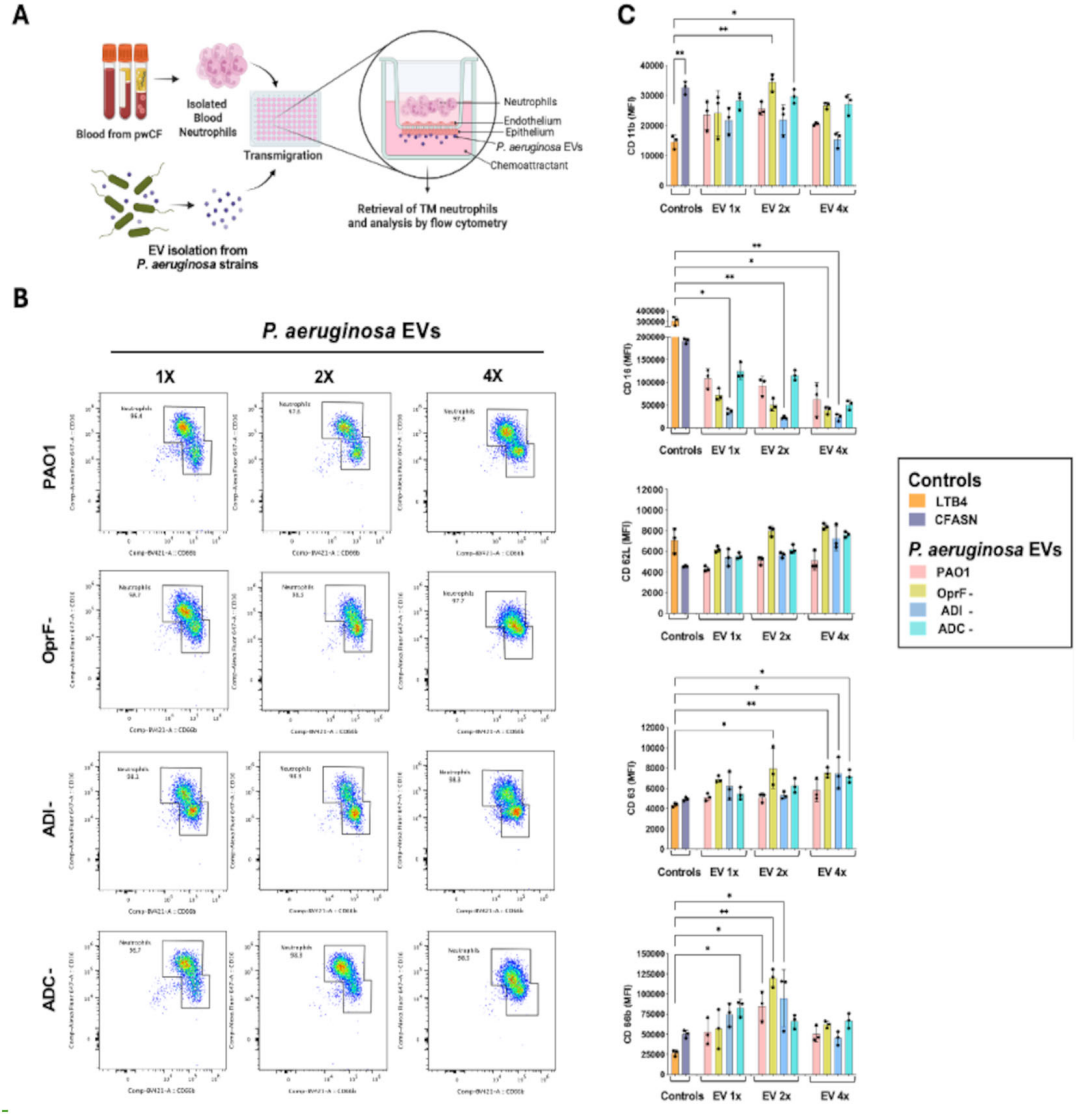
**(A)** Schematic of the experimental protocol. **(B)** Representative 2D plots of CD66b vs. CD16 in live neutrophils transmigrated toward *P. aeruginosa* EVs at 1×, 2×, and 4× (left to right), from PAO1 (wild-type) and mutant OprF−, ADI−, and ADC− strains (top to bottom). **(C)** Bar plots of five phenotypic markers, CD1b, CD16, CD62L, CD63, and CD66b (top to bottom), assessed on lung-conditioned human neutrophils transmigrated to control conditions (LTB4 chemoattractant control and CFASN pathological milieu), vs. *P. aeruginosa* EVs at 1×, 2×, and 4×. * and ** show significant differences at *P* < 0.05 and *P* < 0.01, respectively (*N* = 3 per condition, ANOVA).

EVs isolated from culture of wild-type and mutant strains of *P. aeruginosa* were further assessed for their impact on lung neutrophil conditioning in our biomimetic model of human airway inflammation (Margaroli et al., 2021), using the modified 96-well version to facilitate dose–response testing (Viola et al., 2025). Naive neutrophils purified from the blood of pwCF were transmigrated through a combined human endothelial (HUVEC) and epithelial (H441) barrier in various conditions (**Figure 4A**). Transmigration conditions related to the presence of specific milieu on top of the epithelial layer, namely, CF airway supernatant (CFASN, pathological milieu), leukotriene B4 (LTB4, chemoattractant control), media only, and *P. aeruginosa* EVs (experimental conditions). While LTB4 recruits neutrophils to the lumen in conditions emulating normal airways, CFASN recruits and conditions neutrophils pathologically to adopt an abnormal fate we termed “GRIM” for its excessive granule release, immunomodulatory functions, and metabolic licensing (Margaroli et al., 2021; Forrest et al., 2018).

Prior studies of CF sputum revealed concentrations of EVs of 10^10^ per mL (Wahlund et al., 2017; Mathieu et al., 2019; Useckaite et al., 2020; Trappe et al., 2023), of which 10% (10^9^ per mL) were from *P. aeruginosa* based on proteomics data presented here (**Figure 1**). Since live neutrophils can reach 5 × 10^6^ per mL of CF sputum, we aimed for a concentration of 200 bacterial EVs per neutrophil. Considering a yield of 5 × 10^5^ neutrophils per well in our model, we evaluated lung-recruited neutrophils under three different doses of exposure to *P. aeruginosa* EVs, namely, 2.5 × 10^6^ (50 EVs per neutrophil = 1×), 5 × 10^6^ (100 EVs per neutrophil = 2×), and 10^7^ (200 EVs per neutrophil = 4×) bacterial EVs. To this end, naive blood neutrophils were transmigrated for 14 h through our 96-well endothelial/epithelial platform, toward specific apical conditions (**Figure 4A**). This platform allows for robust retrieval of live neutrophils post-transmigration, which can then be analyzed by flow cytometry (**Supplementary Figure 11**). Representative 2D plots featuring CD66b (secondary granule marker) vs. CD16 (tertiary granule and secretory vesicle marker) are shown in **Figure 4B**. Significant differences between conditions in surface neutrophil expression of CD66b and CD16, as well as other activation markers—CD11b (secondary granule, tertiary granule, and secretory vesicle marker), CD62L (shed upon transmigration), and CD63 (primary granule marker)—are indicated in **Figure 4C**. All conditions showed similar effectiveness at promoting neutrophil transmigration, with no difference in CD62L levels. Neutrophil activation was evident as increased levels of CD11b, CD63, and CD66b and loss of CD16 in multiple conditions including *P. aeruginosa* EVs compared to the LTB4 chemoattractant control were within the effects observed with the CFASN pathological milieu. This activation was more pronounced at 2× and 4× doses than at 1× doses and for EVs originating from mutant strains (notably OprF− and ADI−) than wild-type PAO1.

## Discussion

EVs are nanoparticles produced by cells from all kingdoms, enabling complex crosstalk within multicellular organisms not only among host cell subsets but also between host cells and the microbial species they harbor. Emerging evidence shows that host/microbial interactions are facilitated by the exchange of RNA and protein molecules within EVs (Munhoz Da Rocha et al., 2020). In the CF lung, cooperative crosstalk between the host epithelium, leukocytes, and bacterial species contributes to a non-resolving activation of the host innate immune system in the face of chronic bacterial (and fungal) challenge. Here, we probed for the presence of bacterial EVs in CF airway fluids and studied in more depth the ability of EVs from the common CF microbe *P. aeruginosa* to elicit a response from human epithelial cells and lung-recruited neutrophils.

First, we used MS-based proteomic analysis and computational queries against human and microbial species databases to characterize isolated EVs from BALF and sputum of pwCF and demonstrate that host and microbial EVs do co-exist in those biofluids. As bacterial infections with *S. aureus*, *H. influenzae*, and *P. aeruginosa* are common in pwCF along with infections by the fungus *A. fumigatus* (Lipuma, 2010; Pienkowska et al., 2023), those four organisms were our primary foci in this study. As expected, ~90% of EV proteins identified in BALF (from children) and sputum (from teenagers) were of human origin, and ~10% originated from microbial sources. A higher prevalence and intensity of neutrophil-derived proteins such as neutrophil elastase and myeloperoxidase were observed in BALF clinical samples with increasing age and in sputum samples, as previously reported (Useckaite et al., 2020), confirming the potential of host EV proteins to serve as clinical biomarkers. The overall percentage of *P. aeruginosa* EV proteins identified in sputum was higher than in BALF, likely reflecting the increased age of donors in the former group. Indeed, in CF, an increased prevalence of *P. aeruginosa* infection in teenagers and adults and an increased prevalence of mucoid strains with age have been documented (Heltshe et al., 2018). *P. aeruginosa* EV proteins involved in multidrug resistance (e.g., MexB) and virulence (e.g., components of the type II secretion system) were identified in the sputum of teenagers and adults with CF, suggesting a potential for microbial EV proteins to serve as biomarkers of infection.

After establishing the co-existence of host and microbial EVs in lung fluid from pwCF, we investigated whether EVs from *P. aeruginosa* would impact EV release from patient-derived epithelial cultures. Prior studies showed that *P. aeruginosa* EVs can encapsulate virulence factors and PAMPs like LPS to stimulate host responses in CF, including the secretion of IL-8 from epithelial cells (Bauman and Kuehn, 2006). We noted changes in the concentration of host EVs released from airway epithelial cultures (derived from both children with CF and controls) upon exposure to *P. aeruginosa* EVs, with higher numbers detected in apical cultures from pwCF. Post-*P. aeruginosa* EV exposure, proteomic analysis of apical EVs from stimulated airway epithelial cultures of pwCF showed an enrichment in typical neutrophil-activating molecules (e.g., ICAM-1), as well as histone H4, which has previously been shown to stimulate neutrophil activation through membrane permeabilization (Hsieh et al., 2021). Additionally, a small increase in arginosuccinate synthase, a biosynthetic enzyme in the host arginine pathway, was upregulated in apical airway epithelial EVs post-exposure to *P. aeruginosa* EVs. This is potentially interesting as microbes can employ strategies to modulate host immune responses by interfering with host arginine metabolism (Gogoi et al., 2016), and this further supports the idea of relay signaling by EVs, even across kingdoms (Askenase, 2021).

Reassuringly, 98% of identified proteins in *P. aeruginosa* EVs mapped to PA01 annotated hits, indicating that the CF strain included in our study was indeed PA01-like, in keeping with reports of a high prevalence of PA01-like strains in pwCF (Grace et al., 2022). Enrichment analysis highlighted metabolic proteins within *P. aeruginosa* EVs, including mediators of the arginine pathway, as well as known virulence factors and adhesion proteins such as OprF. These findings are consistent with those of a recently published study identifying differences between the proteome of *P. aeruginosa* cells and secreted EVs, with a significant enrichment in pathogenic and nutrient acquisition proteins in the latter (Zavan et al., 2023).

Using a PA01 mutant library (Held et al., 2012), we examined the impact of exposure to EVs from wild-type *P. aeruginosa* and three select mutant strains on the secretion of human EVs and cytokines by airway epithelial cultures. Specifically, we used secreted EVs from mutant strains with a deletion in OprF, an adhesin bacterial protein known to bind to lung epithelial cells (Azghani et al., 2002), and in two arginine metabolic enzymes, namely, ADI and ADC. In airway epithelial cultures derived from children with CF, a decrease in secretion of apical EVs and IL-8 upon exposure to *P. aeruginosa* EVs from ADI− and ADC− strains was observed. In prior studies (Luckett et al., 2012), *P. aeruginosa* was found to use extracellular arginine as a nutrient for its own growth, providing a fitness advantage when arginine-containing peptides were the sole source of nitrogen in the environment. Our findings suggest that *P. aeruginosa* may transfer arginine pathway enzymes via EVs to the CF lung epithelia, which warrants further investigation. Meanwhile, a significant decrease in IL-8 secretion was observed in the apical fluid from airway epithelial cultures exposed to EVs from OprF− strain compared to wild-type PAO1. However, a similar effect was not observed with epithelial EV release, indicating that some *P. aeruginosa* proteins packaged into EVs may influence host immunity via classical pathways, e.g., NF kappa/IL-8, while leaving EV secretion relatively unscathed.

Importantly, significant changes in the surface profile of lung-conditioned neutrophils produced in our biomimetic model of human airway inflammation were also observed upon exposure to a physiologically relevant burden of *P. aeruginosa* EVs. In particular, we observed significant loss of CD16 and upregulation of CD11b, CD66b, and CD63, which together denote substantial shedding of CD16 and degranulation of all granules (tertiary, secondary, and primary). These are reminiscent of the effect seen with exposure to CF airway fluid, as demonstrated in our prior *in vivo* and *in vitro* studies (Tirouvanziam et al., 2008; Laval et al., 2013; Margaroli et al., 2021). Finally, we observed apparent differences in the effects on lung-conditioned neutrophils provoked by the exposure to EVs from mutant strains (notably OprF− and ADI−) compared to wild-type *P. aeruginosa*.

Taken together, the findings from our study suggest that bacterial EVs found in CF airway fluid influence host epithelial and neutrophil responses. We acknowledge the limitations of this study. First, MS-based proteomics of EVs, as carried out in our pilot study, does not differentiate between proteins present in the intravesicular space from those attached electrostatically to the membrane (“corona”). As highlighted by the International Society for Extracellular Vesicles (Théry et al., 2018), EV-associated functionality can arise from both intravesicular and corona proteins. The corona is more accessible on EVs and may have higher biomarker or therapeutic utility in certain clinical samples, including in CF. Thus, determining whether specific proteins are intravesicular or surface-associated is an important consideration for future studies. Second, although 26 clinical samples were used in **Figure 1**, the number of patient-derived cultures to test bacterial EV challenge in subsequent figures was low, mainly due to a lower number of bronchoscopies performed in the era of CFTR modulators. We also observed patient variability in response to *P. aeruginosa* EVs, and a larger number of samples would be of benefit in future studies. However, these results may be genuinely reflective of variable lung response in CF to pathogens. This pilot study and novel targets identified by MS-based omics may serve as a gateway for future mechanistic studies. Further studies into the mechanisms at play may suggest new ways to counter chronic infection and bacterial tolerance to benefit pwCF.

## Data Availability

All relevant data is contained within the article: The original contributions presented in the study are included in the article/**Supplementary Material**, further inquiries can be directed to the corresponding author/s.

## Supplementary material

Supplementary Table 1 Clinical Characteristics

Supplementary Table 2 Summary multi tab excel of MS IDs (LFQs for BALF and sputum, all species)

Supplementary Table 3 List of apical EV proteins pre/post PA EV stimulation from CF and control and GO analysis in multi tab excel

Supplementary Table 4 List *S. aureus* and *P. aeruginosa* EV proteins and top pathways in multi tab excel
