# Supplementary material for: Interkingdom signaling elicited by bacterial extracellular vesicles in human cystic fibrosis airway epithelium and neutrophils
Source: Front Cell Infect Microbiol. 2026 Mar 2;16:1695102. doi: 10.3389/fcimb.2026.1695102 (PMC12989543; doi:10.3389/fcimb.2026.1695102)
Supplement: Supplementary file 1 [file DataSheet1.pdf]

Supplementary Results

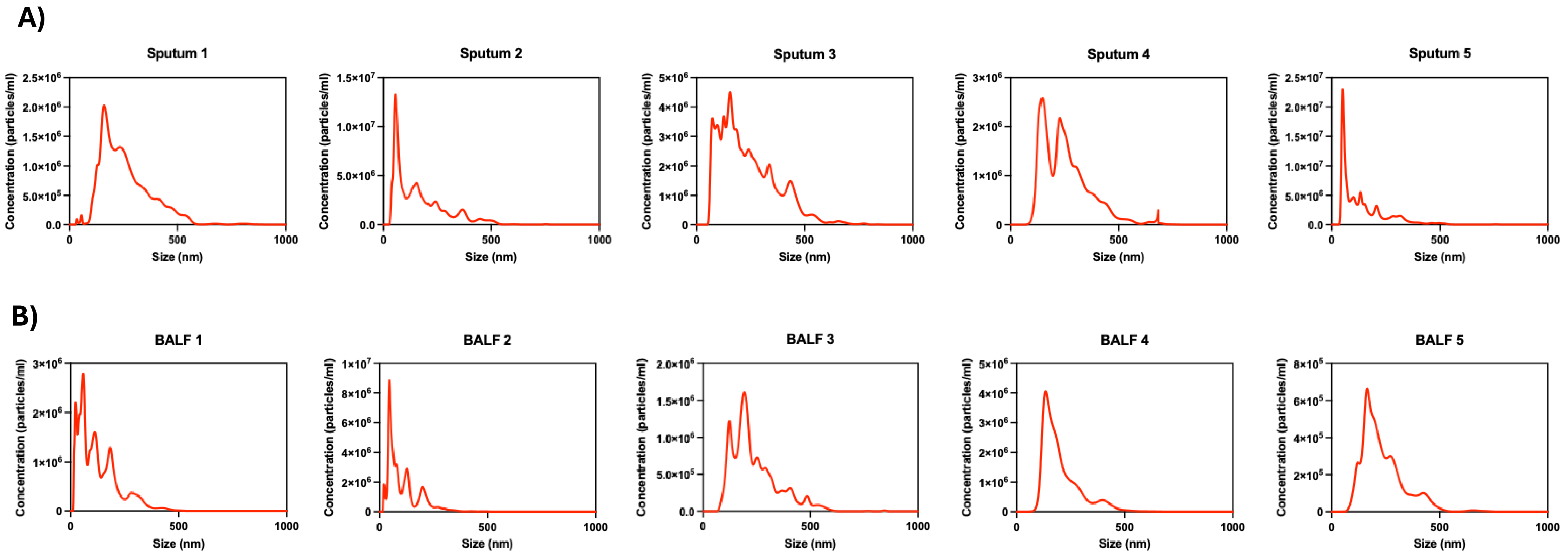

Supplementary Figure 1

A: Concentration and size distribution profile of sputum EV particles from 5 representative pwCF

B: Concentration and size distribution profile of BALF EVs particles from 5 representative pwCF

A

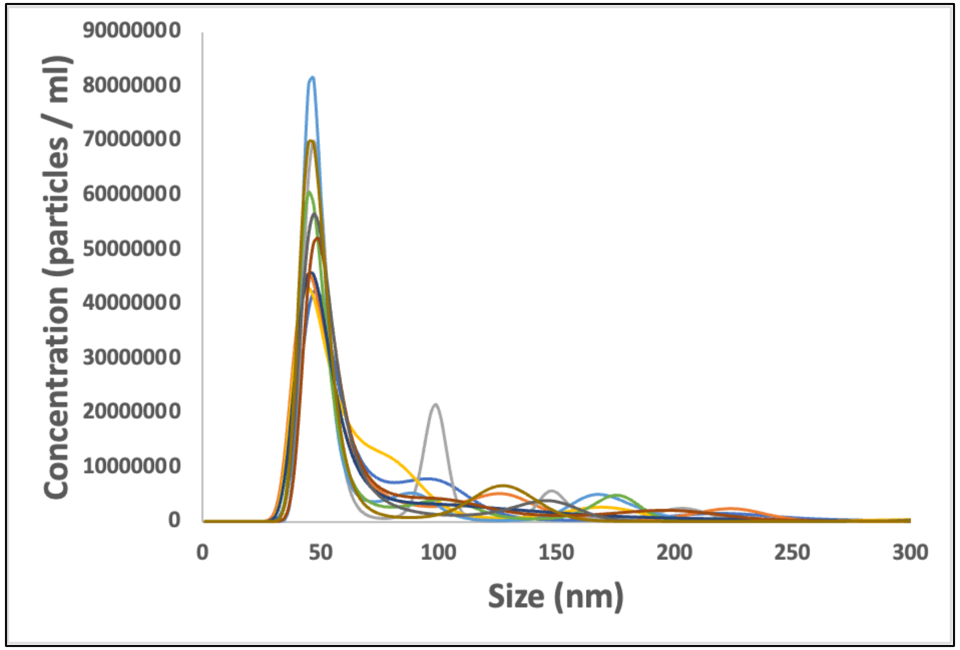

B

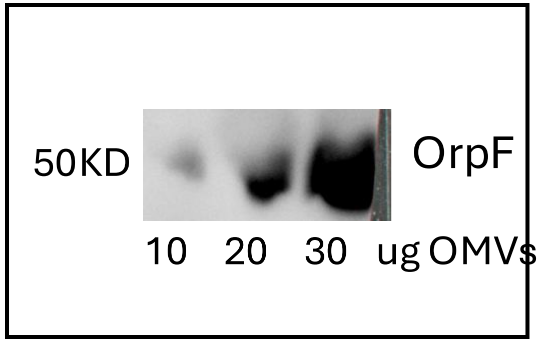

Supplementary Figure 2

A. Concentration and size distribution of EVs isolated from *P. aeruginosa (each trace is a replicate, n=10)*

B. Validation of presence of outer membrane protein OrpF *in P. aeruginosa* EVs.

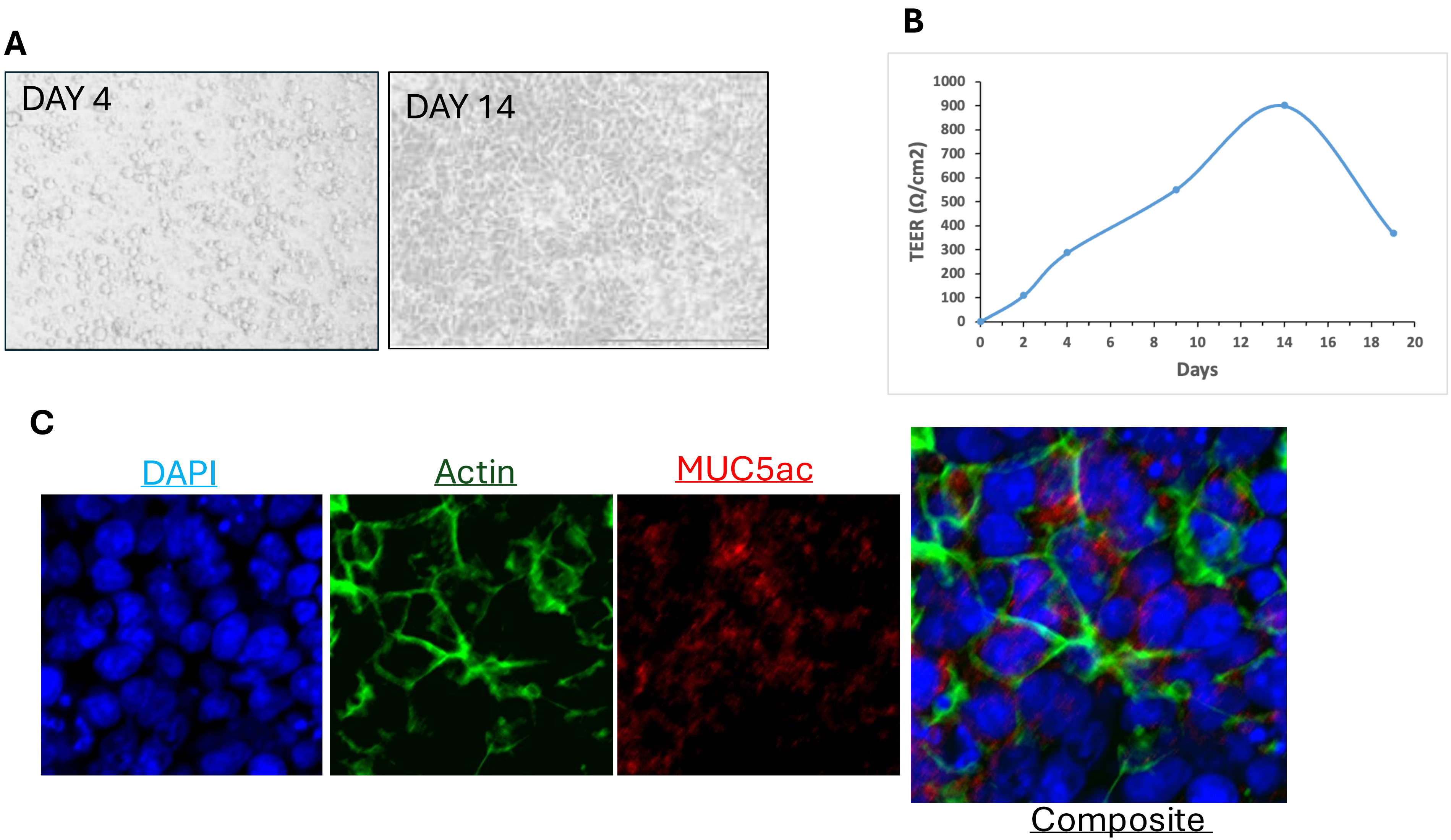

Supplementary Figure 3

A. ALI cultures from a pwCF showing formation of tight junctions on day 14.

B. TEER readings from Day 0-19 for ALI cultures of pwCF.

C. ICC images showing DAPI, MUC5AC, ACTIN of ALI cultured from a pwCF (Day16). (63x magnification)

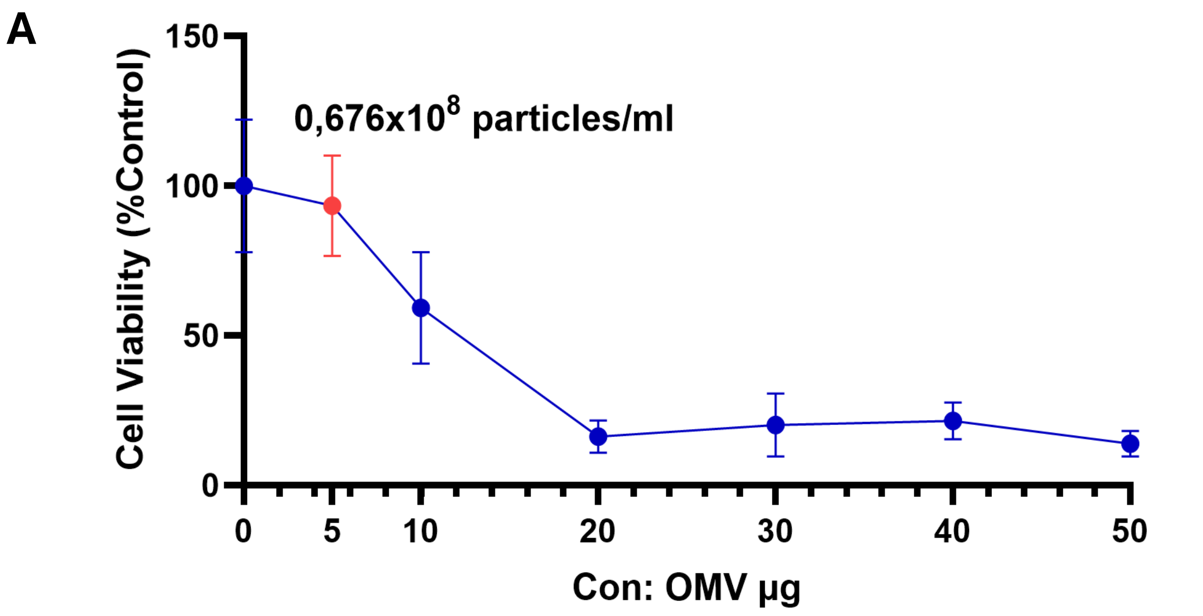

Cell Viability	100	93.3	59.2	16.2	20.1	21.4	13.8
OMV µg	0	5	10	20	30	40	50
Particles/ml	-	$0.676x10^8$	$1.35ex10^8$	$2.704x10^8$	$4.06x10^8$	$5.41x10^8$	$6.76x10^8$

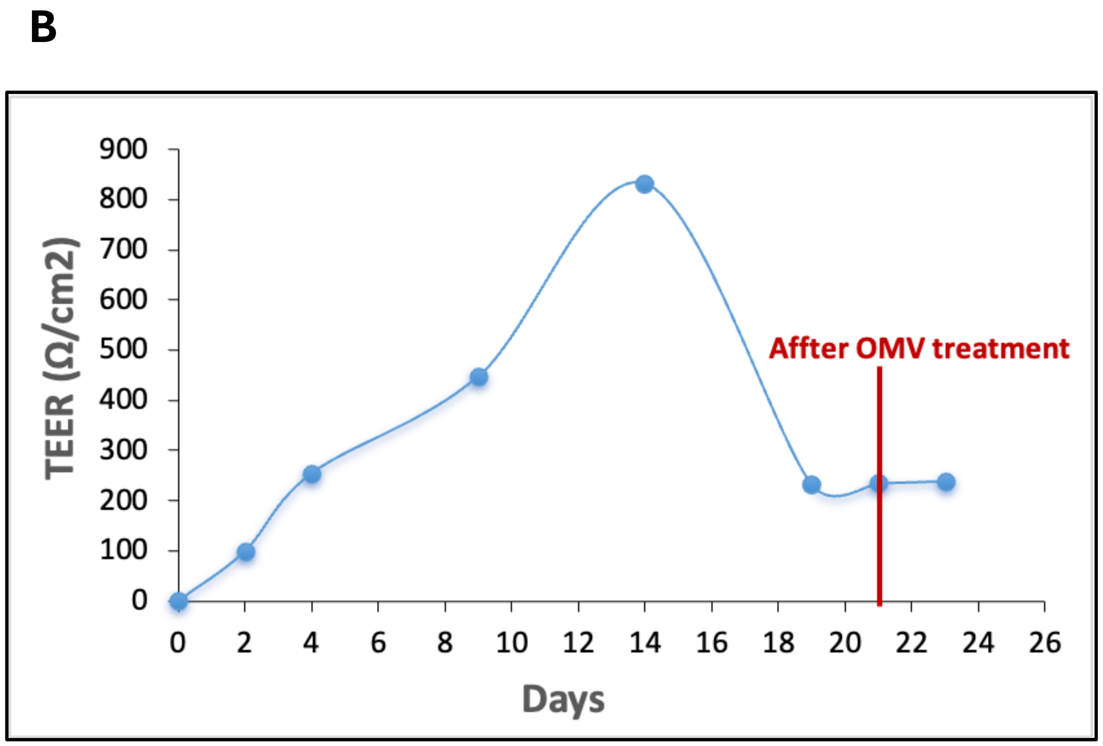

Supplementary Figure 4

A. MTT concentration curve of CF cell viability with increasing concentration of *P. aeruginosa* EVs

B. TEER reading pre and post *P. aeruginosa* EV treatment ~21 days

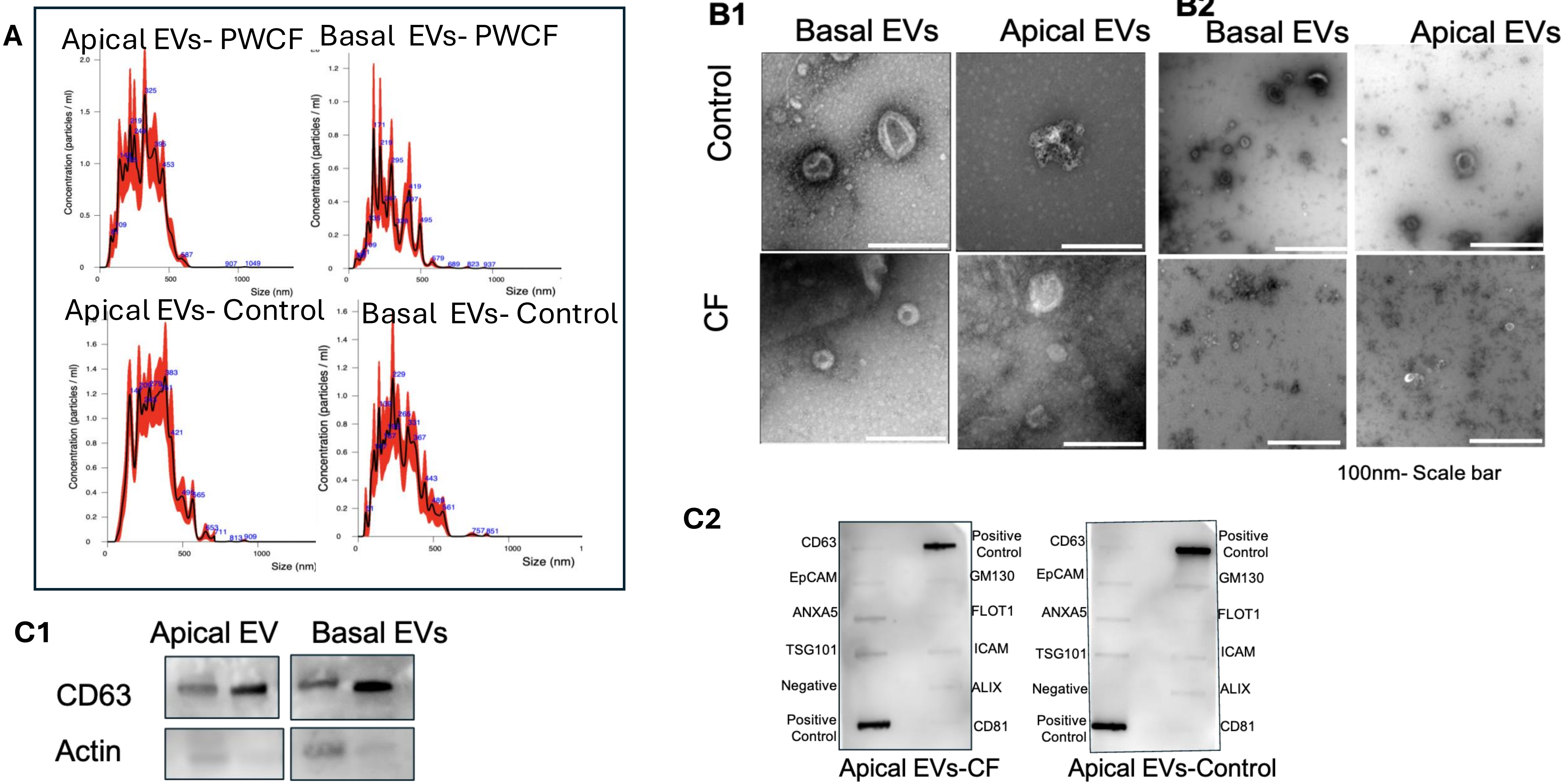

Supplementary Figure 5

A Representative concentration and size distribution graphs for apical and basal EVs from airway cultures of a pwCF and control.

B. Representative TEM Images of apical and basal EVs from airway cultures of pwCF and Control at **1**. 200x and **2**. 40x magnification

C. 1. Western blot images of the EV marker CD63 in apical and basal EVs **2**. Exoarray of markers in apical EVs from PwCF and control

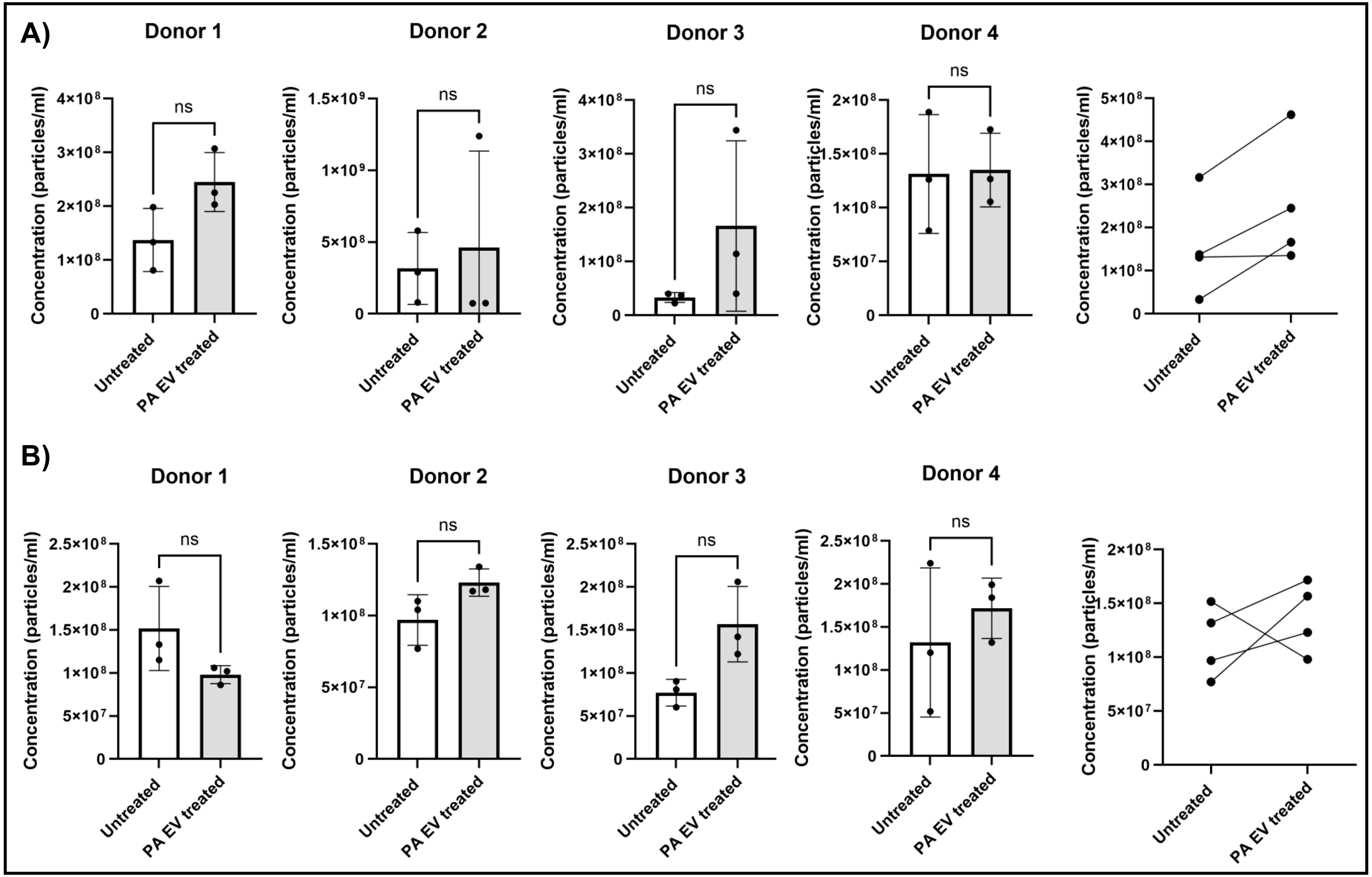

Supplementary Figure 6. Basal EV particle concentration per mL in A) pwCF and B) control samples from four donors after *P. aeruginosa* (PA) EV treatment. Statistical analysis was performed using a paired non-parametric Wilcoxon rank test, and error bars indicate the mean ± SD (n=4).

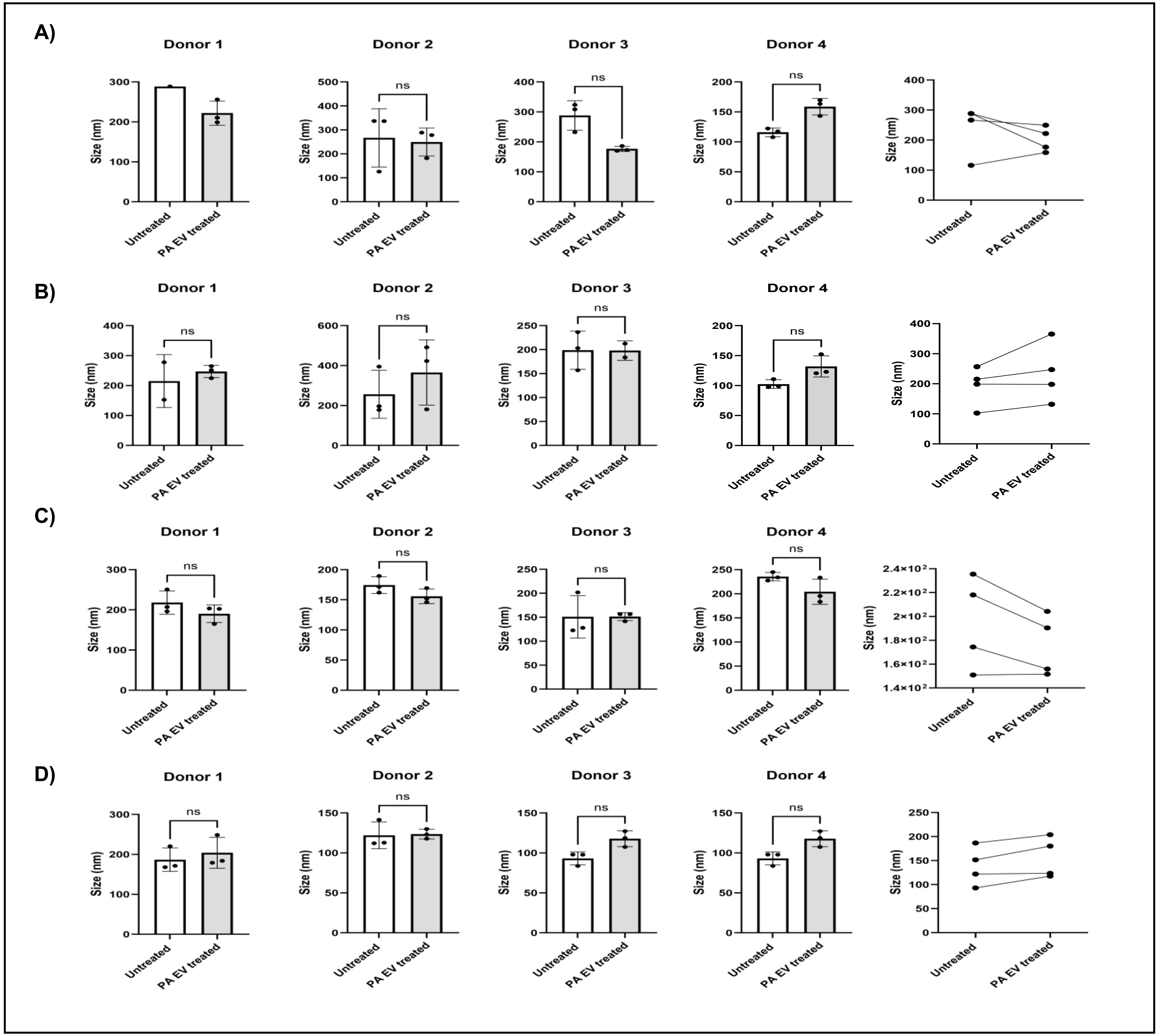

Supplementary Figure 7.
Particle size distribution of EVs in **A)** apical medium from pwCF, **B)** basal medium from pwCF, **C)** apical medium from control samples, and **D)** basal medium from control samples. Statistical analysis was performed using paired non-parametric wilcoxon rank tests, and error bars indicate the mean ± SD (n=4)

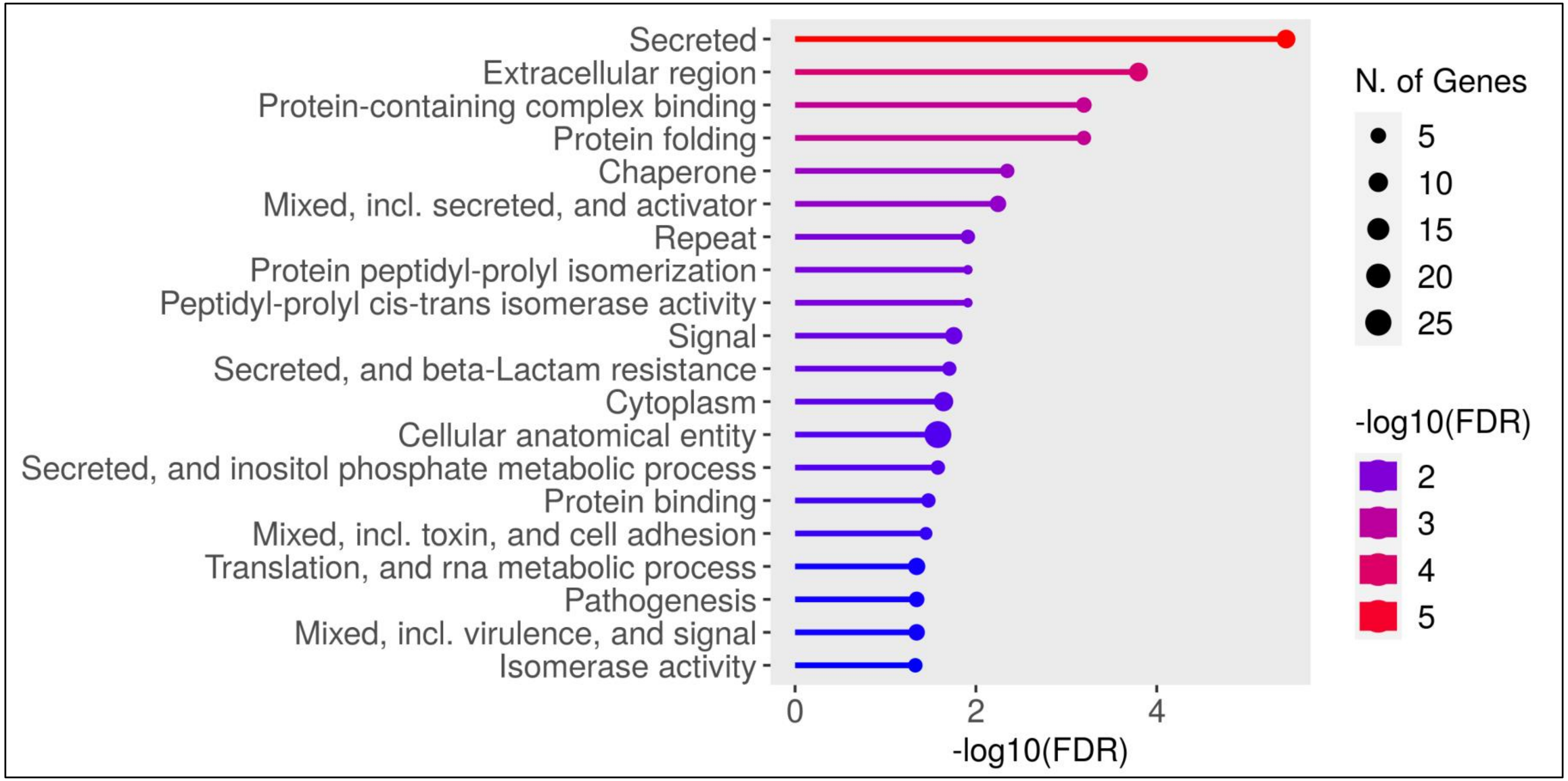

Supplementary Figure 8: EVs were isolated from a clinical CF strain of *S. aureus* as outlined in methods and subjected to MS and bioinformatic analysis . The total proteins identified are in Supplementary Table 4. Significantly enriched GO biological processes (ShinyGO) for *S. aureus* EV proteins are displayed along with corresponding gene number and FDR (inverse log) range.

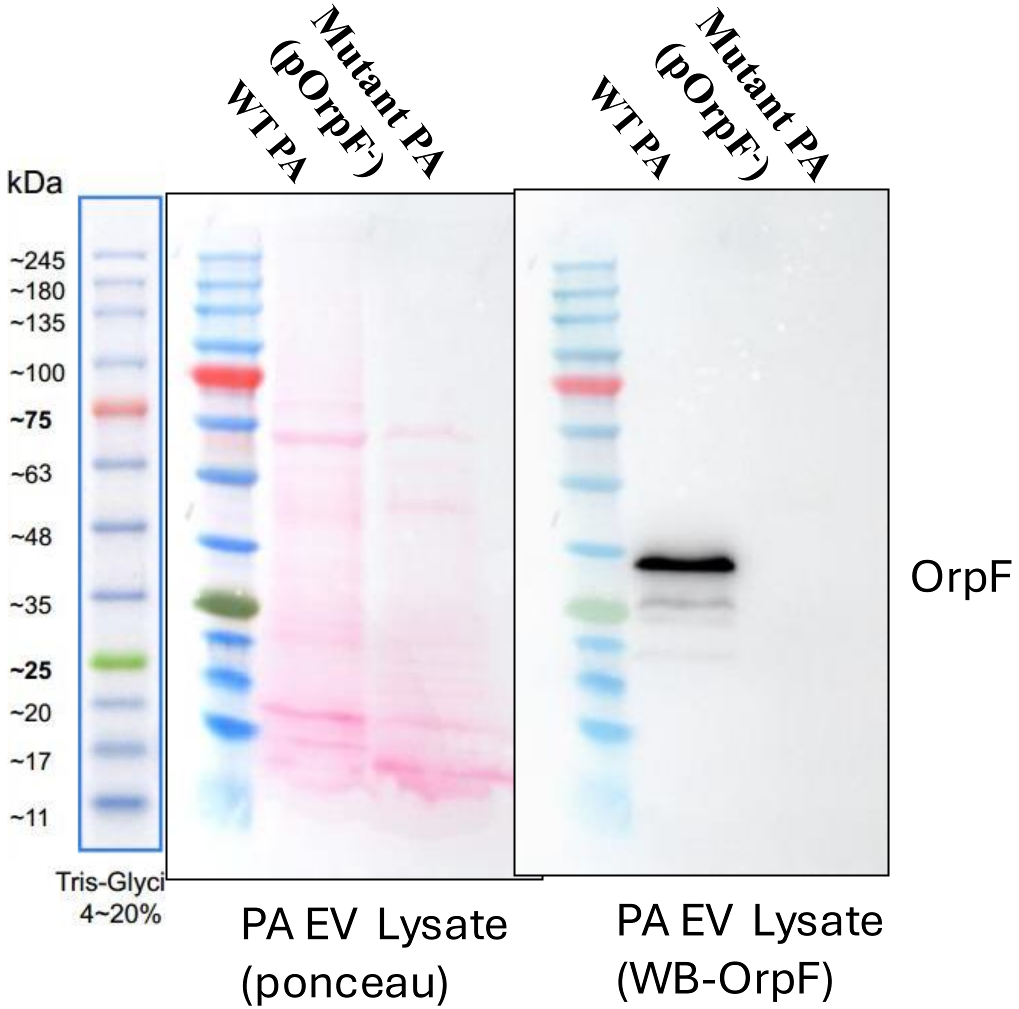

Supplementary Figure 9: WT and mutant EVs isolated from *P. aeruginosa* (PA) WT and mutant p-OrpF strains were lysed and immunoblotted for OrpF to demonstrate OrpF was not expressed in mutant PA (OrpF$^-$) EVs

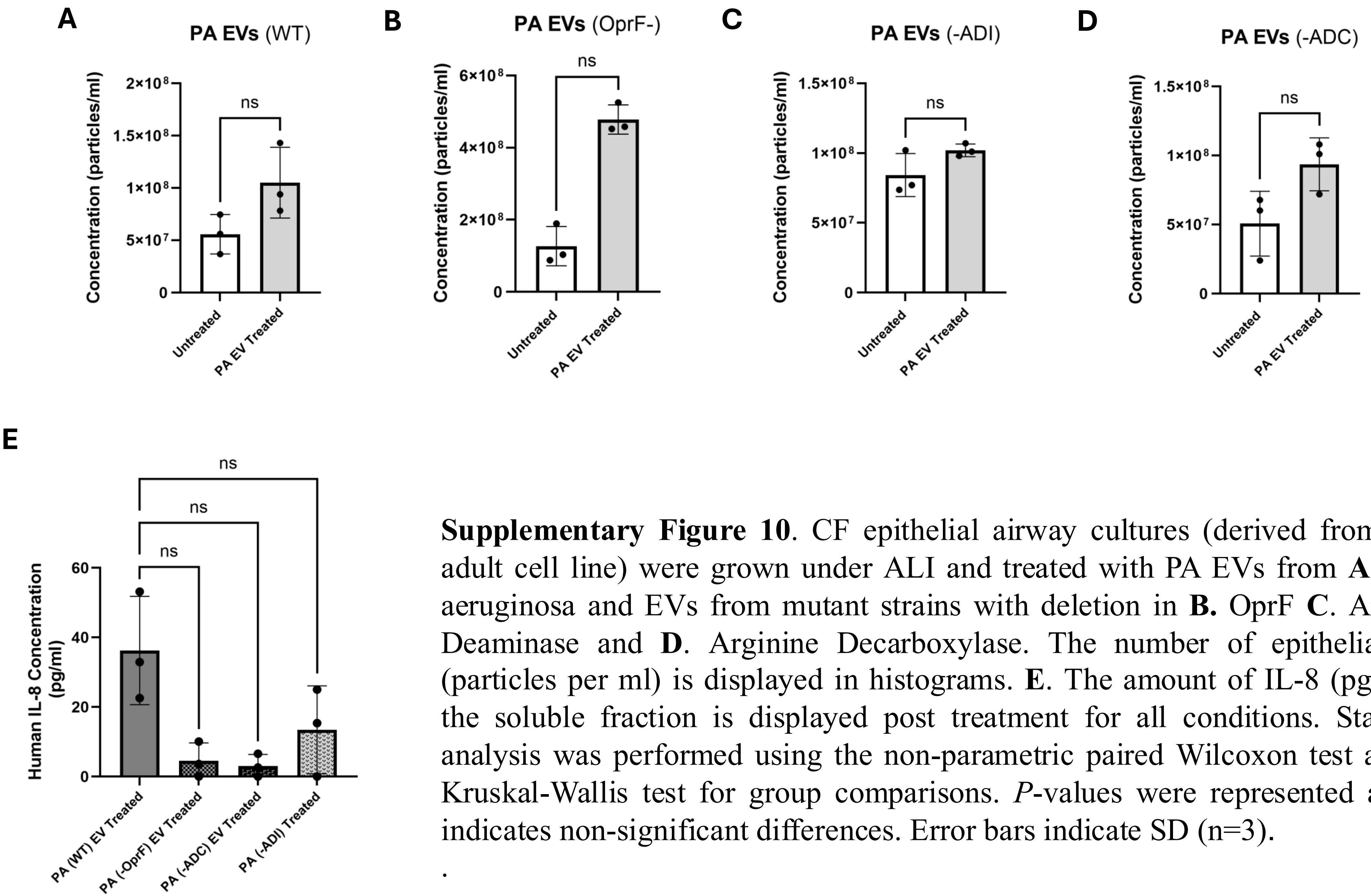

Supplementary Figure 10. CF epithelial airway cultures (derived from a CF adult cell line) were grown under ALI and treated with PA EVs from **A**.WT P. aeruginosa and EVs from mutant strains with deletion in **B.** OprF **C**. Arginine Deaminase and **D**. Arginine Decarboxylase. The number of epithelial EVs (particles per ml) is displayed in histograms. **E**. The amount of IL-8 (pg/ml) in the soluble fraction is displayed post treatment for all conditions. Statistical analysis was performed using the non-parametric paired Wilcoxon test and the Kruskal-Wallis test for group comparisons. *P*-values were represented as 'ns' indicates non-significant differences. Error bars indicate SD (n=3).

.

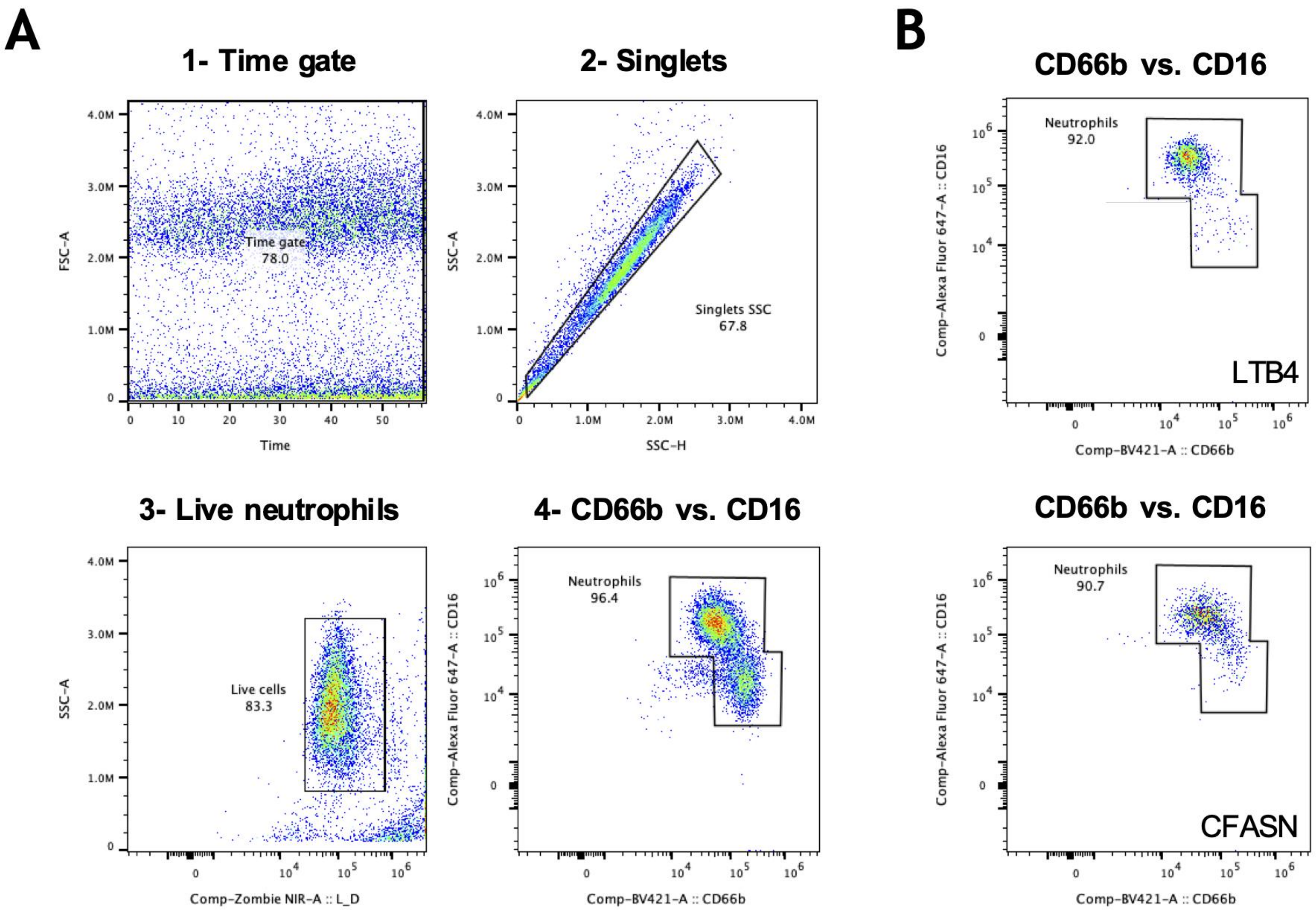

Supplemental Figure 11. A: Gating strategy using time gate (1), singlet gate (2), live neutrophil gate (3) and finally a 2D plot of CD66b vs. CD16. **B:** Neutrophils were transmigrated towards LTB4 (top) or CFASN (bottom), showing higher expression of CD66b and lower expression of CD16 in the latter, as expected (n=3)
