# Supplementary material for: Interkingdom signaling elicited by bacterial extracellular vesicles in human cystic fibrosis airway epithelium and neutrophils
Source: Front Cell Infect Microbiol. 2026 Mar 2;16:1695102. doi: 10.3389/fcimb.2026.1695102 (PMC12989543; doi:10.3389/fcimb.2026.1695102)
Supplement: Supplementary file 2 [file DataSheet2.pdf]

Supplementary Information

Subjects: Bronchial Lavage Fluid (BALF) and brushings were obtained through The Study of Host Immunity and Early Lung Disease in Cystic Fibrosis (SHIELD-CF) or RECOVER (Real World Clinical Outcomes with Novel Modulator Therapy Combinations in People with CF) from children with CF at Children's Health Ireland (CHI) and from adults with CF attending St. Vincent's University Hospital (SVUH). All study participants were recruited as approved by the Ethics Medical Research Committee at CHI or SVUH. Bronchial brushings were obtained via consent from CF and control donors from persons undergoing bronchoscopy (and cultured under air liquid interface) (GEN/807/20) (GEN/228/11)

Cell culture: Bronchial brushings were incubated in PBS (50 µg/ml pen-strep (G1397, Sigma-Aldrich (St. Louis, MO, USA)), 50 µg/ml gentamycin (A2942-20mL, Sigma-Aldrich (St. Louis, MO, USA)), and 1.25 µg/ml amphotericin B (1397-89-3-20mL, Sigma-Aldrich (St. Louis, MO, USA)) for 10 minutes. The PBS was gently agitated with a pipette to detach the cells from the brush. The cells were further pelleted at 500g for 5 min at 4°C and resuspended in PneumaCult™-Ex Plus Medium (05040, STEMCELL Technologies (Vancouver, BC, Canada)), (50 µg/ml pen-strep, 50 µg/ml gentamycin, 1.25 µg/ml amphotericin B)). Resuspended cells were seeded into human collagen coated T25 flask and incubated at 37°C in 5% CO2 in PneumaCult™-Ex Plus Medium, until 70% confluency was achieved by changing media every alternative day. The cells from T25 were trypsinized, counted and seeded at a density of 25 x 104 cells/mL into apical compartment of 6.5 mm CELLTREAT® 0.4 µm Polyethylene Membrane Inserts (100-0997, STEMCELL Technologies (Vancouver, BC, Canada)) containing PneumaCult™-Ex Plus Medium in both apical and basal compartments. The cells were cultured and TEER was assessed (every alternative day, after changing the media, until tight junctions were observed). Once the TEER reached 800-1000 Ω, the cultures was switched to air-liquid interface by exposing apical surface to air and basal media was switched to PneumaCult™-ALI basal Medium (05001, STEMCELL Technologies (Vancouver, BC, Canada)), (50 µg/ml pen-strep, 50 µg/ml gentamycin, 1.25 µg/ml amphotericin B). The cells were cultured in total for about 18-21 days, until TEER reached back to physiological level, 200 Ω.

Mucus secretion was assessed at different timepoints during differentiation by inmunofluorescence. Cells were fixed with 4% paraformaldehyde for 20 mins at room temperature over 6.5 mm CELLTREAT® 0.4 µm Polyethylene Membrane Inserts (Stemcell 100-0997), them the cells were wash 3 times with PBS, permeabilized with 0,3% TritonX-100 in PBS for 10 mins at room temperature, and blocked with 5% BSA for 1h at room temperature. Cells were stained with 1:1000 MUC5AC Antibody (#61193T) CellSignaling Technology, Danvers, MA, USA) in 5% BSA overnight at 4°C followed by 3 washed with PBS. Further cells were stained with 1:1000 Invitrogen Goat anti-Rabbit secondary Antibody, Alexa Fluor™647 (A-21245, Thermo Scientific, Rockford, IL)) in 1% BSA for 1h. Nuclei were stained with DAPI 1µL/ml for 10 mins at room temperature. F-actine was stained with Alexa Fluor 488 Phalloidin (A12379, Thermo Scientific, Rockford, IL)), 1h at room temperature. Cells were washed with PBS mounted on cover slips with mounting media and imaged using a Zeiss LSM700 confocal microscope.

EV isolation. EV fractions were isolated from both the apical and basal chambers and subjected to differential ultracentrifugation. After approximately, 21 days, 200ul media was added to the apical chamber of each well and cell supernatants from both apical and basal chambers were removed, centrifuged at 500g for 10 minutes to remove floating cells, Further supernatants were then ultracentrifuge twice at 120,000 g for 120 minutes 4 °C (XL-70 ultracentrifuge, Beckman-Coulter, Villepinte, France) to pellet the EVs. In order to obtain sufficient EVs for further conditions 3-4 replicates for each condition were combined.

Immunoblotting : EV and cell extracts were prepared in RIPA lysis buffer, mammalian protease inhibitor cocktail, pH 7.4, phosphatase inhibitor cocktail). Protein concentration was determined by the BCA assay (Thermo Scientific, Rockford, IL). Equal amounts of protein were separated by SDS-polyacrylamide gel electrophoresis and transferred onto 0.2mm polyvinylidene fluoride (PVDF) membranes. Membranes were blocked (0.05% Tween 20 and 5% non-fat dry milk or 3% BSA) prior to incubation with primary antibodies. Horseradish-peroxidase-conjugated secondary antibodies were visualized using SuperSignal West Pico or Femto reagents (Thermo Scientific, Rockford, IL). Images were obtained using Vilber Fusion FX imager (Vilber, France). The protocol for the Exo-Check exosome antibody array (Systems Biosciences, CA, USA) was as per manufacturing instructions with ~20ug protein (apical EVs) loaded per condition.

Nanoparticle Tracking Analysis (NTA) of particle size distribution in cellular supernatants and BALF and sputum samples was determined by NTA using a NanoSight NS300 system (Malvern Technologies, Malvern, UK) configured with a 488 nm laser and a high sensitivity scientific CMOS camera. Samples were diluted (cellular supernatants 1:5–1:10, BAL 1:500) in particle-free PBS (Gibco, Waltham, MA, USA) to an acceptable concentration, according to the manufacturer's recommendations. Samples were analysed under constant flow conditions (flow rate = 50). Five successive videos were captured for 30 seconds for each sample. Data were analysed using NTA 3.1.54 software.

Transmission Electron Microscopy: For negative stain microscopy, 10μL 2% uranyl acetate alternative (gadolinium triacetate) (Ted Pella Inc, CA, USA) was aliquoted onto Parafilm (Beemis Company Inc, WI, USA), 5 μL of EV enriched sample was added to the negative stain and covered for 10 minutes incubation/drying. A Formvar/silicon monoxide 200 mesh copper grid (Ted Pella Inc, CA, USA) was placed on the stain/samples and incubated for 1 minute. Excess liquid was removed by blotting. The grid was briefly placed on 10 μL of 2% uranyl acetate alternative (gadolinium triacetate) (Ted Pella Inc, CA, USA). Images were acquired using a H7560 transmission electron microscope (Hitachi High-Technologies Corporation Europe, UK) at 100 kV. To find a magnification that shows as many EVs as possible on a single image with sufficient detail to distinguish EV morphological features, we manually evaluated images at magnifications of 60,000 and 200,000 X magnification. At least 9 images were acquired per sample. Images were analyzed using Image J (https://imagej.nih.gov/ij/)

EVs isolation from *P. aeruginosa* and *S. aureus* strains

P. aeruginosa (PA-01) and *S. aureus* USA300 MRSA clinical strains were streaked on an LB agar plate at 37°C for 24 hours. A single colony bacterial from the agar plate was inoculated with sterile stick and suspended into 15ml of LB liquid growth medium tubes. The tubes were incubated at 37°C on a shaker at 200 rpm for 24 hours. After 24 hours cultures were added into 500 ml of LB media to replicate further. After 24 hours of incubation on a shaker, and upon achieving an OD value of 1.5 at 600nm. Bacteria was pelleted down at 6000g for 15 minutes. Supernatant was filtered in 0.2 um membrane and microbial EVs were pelleted at 100,000g for 1.5 hours at 4°C. Pelleted EV were frozen at -80°C for until further analysis.

Treatments of apical cultures with *P. aeruginosa* EVs

The safe dose of EVs for the treatment of ALI cultures were determined by MTT assay. The EV stocks were added in different quantities (5, 10, 20, 30, 40, and 50 ul) into 96 well plated containing epithelial cells for 24 hours. After 24 hours the media was discarded. 50 μL of serum-free media and 50 μL of MTT solution were added in each well and incubated at 37°C for 3 hours. After incubation, 150 μL of MTT solvent was added into each well. the plated were wrapped up in foil and placed on an orbital shaker for 15 minutes. The absorbance was read at OD=590 nm.

Due to high concentration of EVs in the stock EVs were diluted in each dilution were analysed by NTA to determine the number of EVs. Therefore, based on the cell viability results ALI cultures were treated apically with 0.676e+008 particles/ml of EVs, which was incubated for 24 hours. After 24 hours the supernatant was extracted and frozen for further analysis.

***P. aeruginosa* mutants**

Mutated strains of *P. aeruginosa* were obtained from a mutant library from the Salipante group, University of Washington. Further details on Sequence-Verified Two-Allele Transposon Mutant Library are described (Held *et al,* 2012)

ELISA: The supernatant from *P. aeruginosa* EV treated ALI cultures were collected and centrifuges at 1000 rpm for 10 minutes to remove dead cells. Clear supernatants were diluted to ratio of 1:10 and IL-8 was quantified using AuthentiKine™ Human IL-8 ELISA Kit (KE00275, Proteintech, Rosemont, IL, USA) following manufactures protocol.

Mass Spectrometry: EV samples were dissolved in 8 M urea, 100 mM Tris pH 8.5. Proteins were reduced with DTT and alkylated with IAA. Protein digestion was performed by overnight digestion with trypsin sequencing grade (Promega) resuspended in diluted TFA and stored at 4 °C until MS analysis. Samples were run on a Bruker TimsTOF Pro mass spectrometer connected to a Evosep One chromatography system. Tryptic peptides were resuspended in 0.1% formic acid and each sample was loaded on to an Evosep tip. The Evosep tips were placed in position on the Evosep One, in a 96-tip box. The autosampler is configured to pick up each tip, elute and separate the peptides using a set chromatography method[1]. The mass spectrometer was operated in positive ion mode with a capillary voltage of 1400 V, dry gas flow of 3 l/min and a dry temperature of 180 °C. All data was acquired with the instrument operating in trapped ion mobility spectrometry (TIMS) mode. Trapped ions were selected for ms/ms using parallel accumulation serial fragmentation (PASEF). A scan range of (100-1700 m/z) was performed at a rate of 10 PASEF MS/MS frames to 1 MS scan with a cycle time of 1.89s.

MaxQuant protein identification and quantification

The raw data was searched against the Homo sapiens subset of the Uniprot Swissprot database (reviewed) using the search program MaxQuant (https://www.maxquant.org/) (release 2.6.3.0) using specific parameters for trapped ion mobility spectra data dependent acquisition (TIMS DDA). Each peptide used for protein identification met specific MaxQuant parameters, i.e., only peptide scores that corresponded to a false discovery rate (FDR) of 0.01 were accepted from the Maxquant database search. The normalised protein intensity of each identified protein was used for label free quantitation (LFQ).

Protein /Pathway Analysis The Perseus computational platform (version 1.6.15.0) was used to process MaxQuant results. The spectra were searched using the MaxQuant algorithm against reviewed protein sequenced databases from Uniprot KB(https://www.uniprot.org/uniprotkb) namely Human, *Pseudomonas aeruginosa, Staphylococcus aureus, Haemophilus Infleunzae and Aspergillus Fumigatus.* The identified genes were analysed using GO Enrichment analysis from the Gene Ontology website https://geneontology.org/ which connects to the Panther Classifictaion system and via Shiny GO is a graphical gene enrichment tool which links gene set to functional pathways and cellular processes. Enrichment P-value were derived using the hypergeometric test. To correct for multiple testing, False Discovery Rate (FDR) was calculated using the Benjamini-Hochberg method.

Statistical analysis

All quantified data are presented as the mean ± SD for at least three independent experiments. For each experiment, statistical tests are indicated in the results section. Analysis was conducted using Prism 9 (GraphPad Prism, San Diego, CA, USA). Paired testing was used for pre/post *P. aeruginosa* EV treatment with a non-parametric Wilcoxon rank test was used to allow for small samples number without the assumption of normal distribution. A one way non-parametric Kruskal-Wallis test was used for analysis featuring three or more groups.
